# Vaccine-mediated protection against *Campylobacter*-associated enteric disease

**DOI:** 10.1126/sciadv.aba4511

**Published:** 2020-06-24

**Authors:** Benjamin K. Quintel, Kamm Prongay, Anne D. Lewis, Hans-Peter Raué, Sara Hendrickson, Nicholas S. Rhoades, Ilhem Messaoudi, Lina Gao, Mark K. Slifka, Ian J. Amanna

**Affiliations:** 1 Najít Technologies Inc., Beaverton, OR, USA.; 2 Division of Comparative Medicine, Oregon National Primate Research Center, Oregon Health and Science University, Beaverton, OR, USA.; 3 Division of Neuroscience, Oregon National Primate Research Center, Department of Molecular Microbiology and Immunology, Oregon Health and Science University, Beaverton, OR, USA.; 4 Department of Molecular Biology and Biochemistry, University of California Irvine, Irvine, CA, USA.; 5 eBiostatistics Shared Resource, Knight Cancer Institute, 3181 SW Sam Jackson Park Rd., Portland, OR, USA.; 6 Bioinformatics and Biostatistics Core, Oregon National Primate Research Center, Oregon Health and Science University, Beaverton, OR, USA.

## Abstract

*Campylobacter coli* and *Campylobacter jejuni* are responsible for 400 million to 500 million cases of enteric disease each year and represent the most common cause of bacterial gastroenteritis worldwide. Despite its global importance, *Campylobacter* vaccine development has been hampered by the lack of animal models that recapitulate human disease pathogenesis. Here, we describe a naturally occurring *Campylobacter*-associated diarrhea model in outdoor-housed rhesus macaques. Using this model, we developed novel next-generation H_2_O_2_-based *Campylobacter* vaccines that induced strong antibacterial antibodies to multiple *Campylobacter* proteins including flagellin and provided up to 83% protection against severe *C. coli*–associated diarrhea. Whole-genome sequencing of circulating *Campylobacter* strains revealed little to no homology within lipooligosaccharide or capsular polysaccharide loci with the *Campylobacter* vaccine strains used in these studies, indicating that vaccine-mediated immunity was not restricted to a single homologous serotype. Together, these results demonstrate an important advance in vaccine development and a new approach to reducing *Campylobacter*-associated enteric disease.

## Teaser

We describe a novel vaccine that provides protection against *Campylobacter*-associated diarrhea in a natural exposure model.

## INTRODUCTION

*Campylobacter* species cause millions of cases of bacterial gastroenteritis per year and represent one of the most important classes of human pathogens contributing to diarrheal disease throughout the world (*1*). Human disease occurs via a fecal-oral route of transmission and affects both high- and low-income countries. The medical importance of this pathogen was highlighted in a prospective study involving >22,000 children from seven developing countries in which *Campylobacter* rated among the top three causes of moderate to severe diarrhea in 24- to 59-month-old children (*1*). The economic impact of *Campylobacter-*associated disease can be substantial, with annual costs in the United States alone estimated at up to $5.6 billion (*2*). Several *Campylobacter* species have been identified that cause enteric disease, although *Campylobacter jejuni* and *Campylobacter coli* are considered the most important pathogens within this genus and account for the majority of all *Campylobacter*-associated human diseases. Although *C. jejuni* is the most commonly identified cause of *Campylobacter*-associated disease, outbreaks in Peru and Egypt have indicated that *C. coli* is responsible for up to 30 to 37% of *Campylobacter*-associated diarrhea (*3*, *4*). Additional studies have demonstrated a strong correlation between *Campylobacter* burden and childhood growth faltering (*3*), indicating that *Campylobacter* may be a key factor driving poor childhood growth and development outcomes in low resource settings. Together, these studies underscore why this pathogen is recognized as one of the most important global threats in need of targeted vaccine development.

Despite a clear medical need, there is currently no *Campylobacter* vaccine available for use in humans. One of the primary roadblocks has been the lack of robust and reproducible experimental models (*5*). While human challenge models represent the most direct approach (*6*), this is not readily amenable to the experimental manipulation available with animal models. Here, we explored an environmental exposure model of *Campylobacter* infection in rhesus macaques (RMs) to test the efficacy of potential vaccine candidates. Outdoor-housed RM at the Oregon National Primate Research Center (ONPRC) experience a spectrum of acute and recurrent *C. jejuni–* and *C. coli*–associated diarrheal disease that mimics several aspects of *Campylobacter*-associated disease found among human populations that suffer from conditions of poor sanitation and hygiene, including (i) dysbiotic microbiomes, (ii) symptomatic and asymptomatic carriage, and (iii) higher disease incidence rates among infants with a concomitant increase in disease severity compared with adults (*7*, *8*).

We have previously reported on the use of a hydrogen peroxide (H_2_O_2_)–based vaccine platform that is protective against a number of acute and chronic viral infections (*9*). Here, we have adapted this approach to develop novel inactivated *C. coli* and *C. jejuni* vaccines to protect against an enteric bacterial pathogen. Certain strains of *Campylobacter* express lipooligosaccharide (LOS) that appear to be ganglioside mimics (*10*) and to mitigate potential safety issues associated with molecular mimicry, we used vaccine strains of *Campylobacter* [*C. coli* (NTICC13) and *C. jejuni* (CG8421) (*6*)] that lack *neuA*, *neuB*, *neuC*, and *cst* (sialyltransferase) and are genetically incapable of producing ganglioside mimics (*6*). H_2_O_2_-inactivated *Campylobacter* vaccination induced an immunodominant antibody response to bacterial flagellin and provided protective immunity against clinical diarrheal disease in a robust nonhuman primate (NHP) model of naturally occurring *C. coli* infection despite demonstrating little to no homology within the LOS or capsular polysaccharide (CPS) loci compared to circulating *C. coli* strains. In contrast to LOS and CPS, the flagellin genes were highly conserved between the *C. coli* vaccine strain and the circulating strains of *C. coli*, suggesting that this may be a potential target antigen capable of providing antibacterial immunity across disparate *Campylobacter* serotypes. These studies not only demonstrate the feasibility of using this natural challenge model but also provide an important proof-of-concept to support the continued development of novel antibacterial vaccines to prevent *Campylobacter*-associated enteric disease in humans.

## RESULTS

### Diarrheal burden among outdoor-housed RMs

**Fig. 1:**
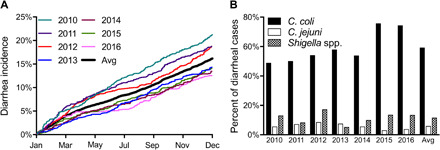
RMs demonstrate consistent acquisition rates of diarrhea with a high *Campylobacter* burden. (**A**) Diarrhea rates were collected for RM from 2010 to 2016 using an electronic health record system. To determine incidence rates, only the first instance of clinical diarrhea for any given animal was counted in each calendar year. These unique diarrheal episodes were divided by the average of 1183 ± 44 animals (±SD) in outdoor sheltered group housing each year. (**B**) For each diarrheal episode (primary or repeat cases), bacterial cultures were tested for the indicated enteric pathogens, *C. coli*, *C. jejuni*, and *Shigella* spp.

Most of the ~5000 NHP at the ONPRC are RM, with approximately 75% of the animals housed outdoors in either 1-acre corral breeding groups or smaller shelter breeding groups (*7*). Diarrheal illness is a substantial concern for captive RM (*7*, *8*), and prior studies have shown that animal groups housed in smaller group shelters are particularly prone to high rates of enteric disease (*7*). Approximately 80% of RM infants are colonized with *Campylobacter* spp. by 1 month of age, and 69 to 97% of juveniles and adults in the outdoor small breeding groups remain clinically asymptomatic carriers of *C. coli* and *C. jejuni* with preliminary unpublished histological evidence indicative of environmental enteropathy. While most animals appear healthy, approximately one quarter of infants will develop acute diarrhea, and half of these animals will progress to chronic/relapsing diarrhea and potentially lethal enteric disease requiring humane euthanasia (*7*). Similar to humans, enteric disease associated with *C. coli* or *C. jejuni* is comparable in these animals, and RM infants and juveniles have higher rates of diarrhea compared to adults (*7*). RM infants also have more severe disease and weight loss compared to the older age groups with chronic diarrhea cases, resulting in a mortality rate that is nearly double that observed among adults (*7*). To expand on these studies, we examined the annual rates of diarrheal incidence in shelter-housed RM from 2010 to 2016 (Fig. 1A). Cumulative annual diarrhea incidence that required hospitalization averaged 16.2 ± 3.4% (mean ± SD). Hospitalization refers to the animals that were removed from the group and housed indoors in a veterinary clinic for 1 or more days to receive appropriate diagnostics and treatment for diarrheal illness and dehydration, often including antibiotics and intravenous fluid therapy. When animals were hospitalized due to diarrhea, fecal samples were collected and tested for the presence of pathogenic bacteria, with a focus on *Campylobacter* and *Shigella* spp. (Fig. 1B). *C. coli* was the most common pathogen associated with diarrhea with an incidence of 59 ± 11% of diarrheal cases followed by *Shigella* (12 ± 4.0%) and *C. jejuni* (5.9 ± 2.0%). Similar to humans, chronic diarrheal disease associated with *Campylobacter* in RM resulted in characteristic histopathologic findings in the large intestine including mucosal hyperplasia, separation of glands by large numbers of lymphocytes and plasma cells, neutrophilic infiltration, decrease in goblet cell numbers, and superficial enterocyte erosion and atrophy (fig. S1). In total, our analysis showed a consistently high burden of *C. coli* and *C. jejuni* among outdoor-housed RM, providing the opportunity to perform *Campylobacter* vaccine field studies under natural fecal-oral exposure conditions.

### Campylobacter vaccine development

**Fig. 2:**
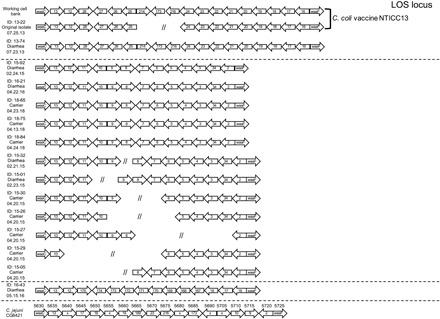
LOS loci demonstrate extensive diversity among RM *C. coli* isolates. Genome sequencing was performed for 15 primary *C. coli* isolates, as well as the *C. coli* vaccine working cell bank. Sequences were queried for the presence of the LOS loci as defined by the *waaC* and *waaF* genes. Genes were compiled and categorized into groups based on sequence similarity (≥80% identity), resulting in 39 groups for the LOS. Isolates with partial sequence results are indicated by //. The LOS genomic regions for the *C. jejuni* CG8421 vaccine strain are also shown. The numbers above each arrow refer to the locus tags in the published sequence (GenBank: CP005388.1), while the numbers within the arrows indicate genes with high similarity (BLAST score > 200) to genes found among the *C. coli* isolates, with gene segments not meeting this threshold indicated with an “x.” Sample isolation dates are provided under each animal ID number. Horizontal dashed lines indicate that there are three distinct strains of *C. coli* based on genetically similar LOS loci. Note that the strains of *C. coli* that circulated in 2015 and 2016 during the vaccine field trials were a mismatch to the *C. coli* and *C. jejuni* vaccine strains used in these studies. Genome sequence accession numbers are provided in the Data and materials availability section.

**Fig. 3:**
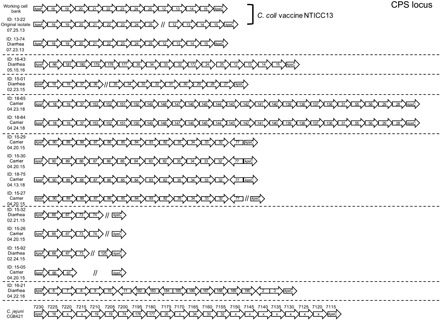
CPS loci demonstrate extensive diversity among RM *C. coli* isolates. Genome sequencing was performed for 15 primary *C. coli* isolates, as well as the *C. coli* vaccine working cell bank. Sequences were annotated (http://rast.nmpdr.org/) and queried for the presence of the CPS loci as defined by the *kpsF* and *kpsC* genes. Genes from all isolates were compiled and categorized into groups on the basis of high sequence similarity (≥80% identity), resulting in a total of 99 groups for the CPS. The genomic organization for each isolate is based on this clustering approach. Isolates with partial sequence results are indicated by //. On the basis of loci organization similarities, isolates were further sorted as shown. For comparison, the CPS genomic regions for the *C. jejuni* CG8421 vaccine strain are also shown. The numbers above each arrow refer to the locus tags in the published sequence (GenBank: CP005388.1), while the numbers within the arrows indicate genes with high similarity (BLAST score > 200) to genes found among the *C. coli* isolates. Gene segments from the *C. jejuni* CG8421 vaccine strain that did not meet this threshold are indicated with an x. The date of sample isolation is provided under each animal ID number. Horizontal dashed lines indicate that there are seven distinct strains of *C. coli* based on genetically similar CPS loci. Note that the strains of *C. coli* that circulated in 2015 and 2016 during the vaccine field trials were a mismatch to the *C. coli* and *C. jejuni* vaccine strains used in these studies. Genome sequence accession numbers are provided in the Data and materials availability section.

Many strains of *Campylobacter* will coexist under hyperendemic conditions since natural infection often does not induce sterilizing immunity in humans or macaques. Successive rounds of reinfection of NHP by multiple strains of *C. coli* and *C. jejuni* have also been observed (*11*), with one study of pig-tailed macaques (*Macaca nemestrina*) finding 10 serotypes of *Campylobacter* among 69 isolates with a mean of 8.3 ± 2 different *Campylobacter* strains identified per infant (*11*). Isolation of multiple *Campylobacter* species and serotypes has also been described in humans (*12*). On the basis of these studies, it was likely that there were multiple strains of *C. coli* and *C. jejuni* cocirculating among the outdoor-housed primates at ONPRC. We performed whole-genome sequencing (WGS) of banked *C. coli* isolates from 2015, 2016, and 2018 and compared them to the vaccine strain isolated in 2013 (fig. S2). We identified three distinct *C. coli* strains on the basis of their LOS loci (Fig. 2) and seven distinct strains on the basis of their CPS loci (Fig. 3). Given the burden of diarrhea observed in shelter-housed animals (Fig. 1A) and the substantial negative impact of diarrheal disease on animal health (*7*, *8*), we initiated a vaccine development program with an initial focus on *C. coli* (i.e., H_2_O_2_-Campy_C_) since it represented the most common enteric pathogen encountered among hospitalized RM cases (Fig. 1B).

**Fig. 4:**
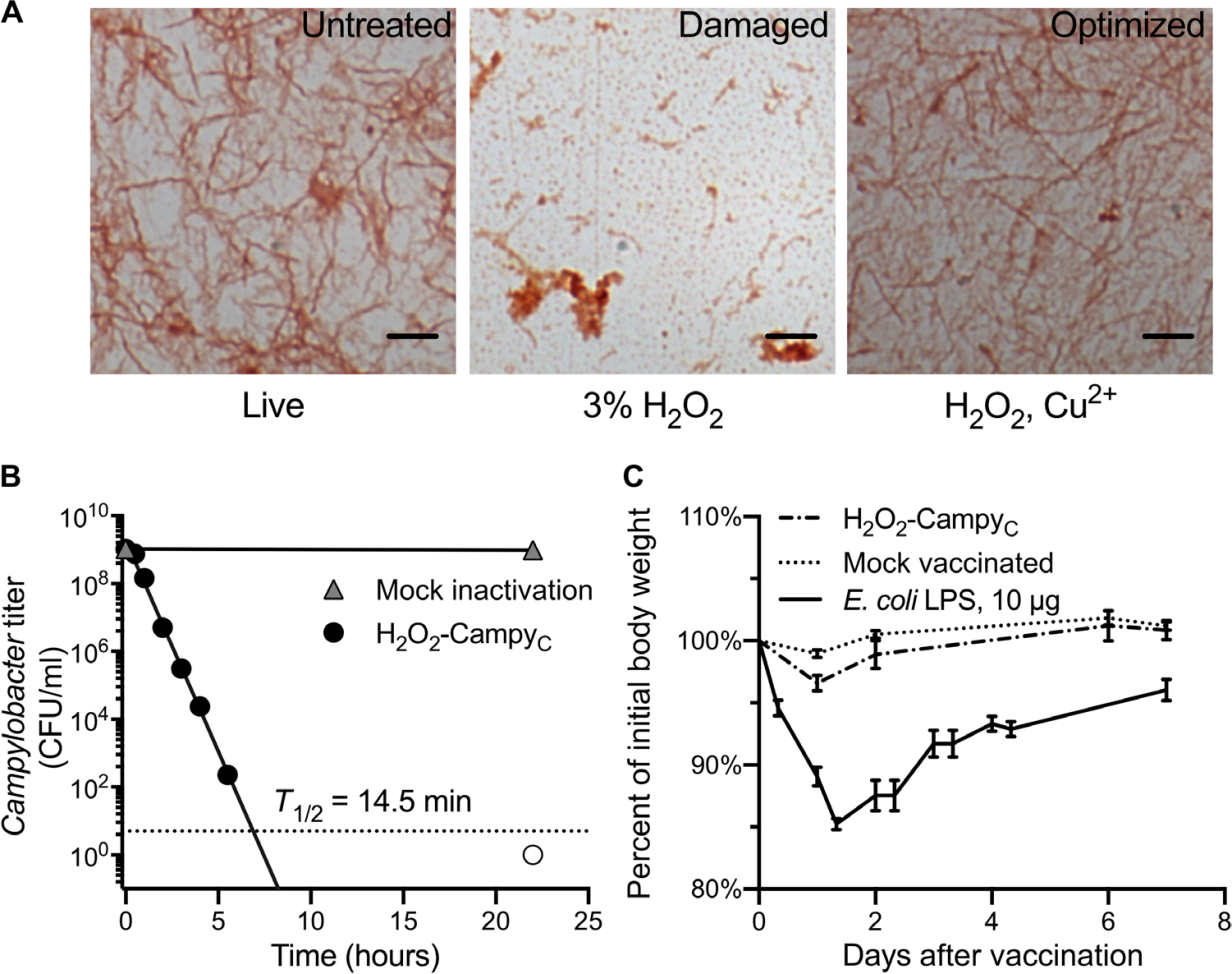
Optimization of H_2_O_2_-based inactivation platform for *Campylobacter* vaccine development. (**A**) Samples of *C. coli* were left untreated (live), treated with a 3% H_2_O_2_ solution for 5 hours (damaged), or treated with an optimal dual-oxidation approach for 22 hours (optimized). Samples were safranin stained and visualized. Scale bar, 5 μm. (**B**) Bacterial inactivation kinetics were performed using an optimized oxidation approach. Samples were collected at the indicated time points and tested for residual live bacteria. At the final time point of 22 hours, no live bacteria were detectable as indicated by the open symbol. A mock inactivation control was followed for the same period of time as shown. Inactivation kinetics are representative of three replicates. (**C**) Mice received the H_2_O_2_-Campy_C_ vaccine, mock vaccine containing alum, or 10 μg of *E. coli* 0111:B4 LPS by the intraperitoneal route. The data represent average weights (±SEM) from two experiments for the H_2_O_2_-Campy_C_ and mock-vaccinated groups (seven animals total per group), and the *E. coli* LPS data are based on one experiment with four mice.

For these studies, we selected a fully sequenced *C. coli* clone that lacked the genes, *neuA*, *neuB*, *neuC*, and *cst* (sialyltransferase), but still showed typical smooth, convex white colonies on blood-agar plates and a Gram-negative stain spiral morphology by microscopy (Fig. 4A, left). Initial inactivation studies using 3% H_2_O_2_ resulted in substantial structural damage and cellular lysis of purified *C. coli* (Fig. 4A, middle). Bearing in mind that microaerophilic bacteria might be more susceptible to oxidative damage, we developed an advanced inactivation approach involving a Fenton-type reaction, in which copper [ cupric ion (Cu^2+^)] is used as a catalyst in an oxidation reaction performed in the presence of lower, less damaging concentrations of H_2_O_2_. Following preliminary small-scale development studies, an optimized approach that maintained the structural integrity of the bacteria was established (Fig. 4A, right). We performed inactivation kinetics by testing samples at regular intervals after incubation with H_2_O_2_ + Cu^2+^ and found rapid loss of bacterial viability with a half-life (*T*_1/2_) = 14.5 min (Fig. 4B). Alum was selected as the vaccine adjuvant on the basis of its established clinical safety record and prior efficacy in conjunction with the H_2_O_2_-based inactivation platform (*9*). To examine general reactogenicity before performing NHP experiments, we conducted small-scale studies in BALB/c mice using a full NHP dose of vaccine that was equivalent to ~300-fold higher dose than that planned for the subsequent RM studies on a milligram per kilogram basis (Fig. 4C). Mice that received the H_2_O_2_-Campy_C_ vaccine were comparable to mock-vaccinated animals (alum alone) and demonstrated no measurable signs of acute weight loss following vaccination. In contrast, mice that received 10 μg of *E. coli*–derived lipopolysaccharide (LPS) as a positive control demonstrated rapid, albeit transient, weight loss indicative of endotoxin-induced reactogenicity. We then conducted a safety study in RM, vaccinating two adult females with the H_2_O_2_-Campy_C_ vaccine and two with alum-only mock vaccines. Health metrics, including body weight, attitude, appetite, urine output, stool quality/quantity, temperature, and vaccine site, were monitored daily for 14 days and no differences were noted between the vaccinated and unvaccinated pairs. Together, these studies indicated that the H_2_O_2_-Campy_C_ vaccine had an acceptable safety profile for further preclinical assessment and we proceeded to immunize RM to determine vaccine efficacy (VE) in a large-scale field study.

### Immune responses in vaccinated RMs

**Fig. 5:**
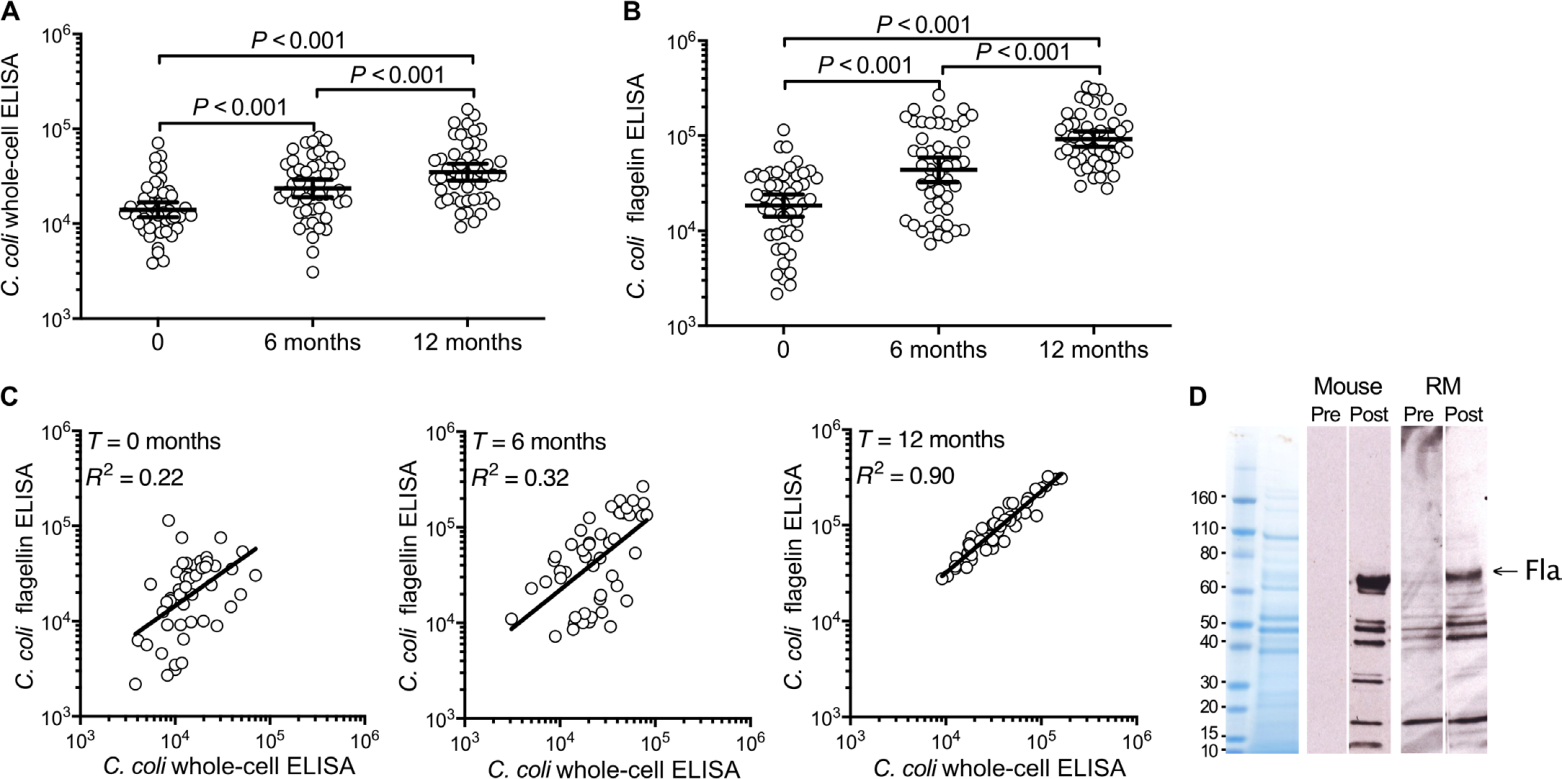
H_2_O_2_-inactivated *Campylobacter* vaccination elicits broad immunity against flagellin and other bacterial antigens. RMs were immunized with the H_2_O_2_-Campy_C_ vaccine at 0 and 6 months with serum collected at 0, 6, and 12 months. Serum antibody titers were determined for (**A**) *C. coli* whole-cell lysate and (**B**) purified *C. coli* flagellin. Geometric means with associated 95% CIs are shown for each time point. Statistical comparisons were made on logarithm transformed titers using repeated measures ANOVA with Tukey’s multiple test correction. (**C**) Titers were matched by individual animals and compared at 0, 6, and 12 months. A log-log linear regression was performed for each time point with the *R*^2^ coefficient of determination shown. (**D**) *C. coli* whole-cell lysates were analyzed by SDS–polyacrylamide gel electrophoresis (SDS-PAGE) and subsequent Western blot. SDS-PAGE lane 1, molecular weight markers; lane 2, Coomassie-stained *C. coli* lysate. Lanes 3 to 6 represent Western blots with mouse preimmune serum (lane 3), mouse postimmunization serum (lane 4), RM preimmune serum (lane 5), and RM postimmune serum (lane 6). Fla, flagellin protein band.

We initiated NHP vaccination studies by immunizing animals from outdoor breeding groups with the H_2_O_2_-Campy_C_ vaccine candidate, with 60 animals that were monitored for diarrheal disease for up to 1 year after primary vaccination. There were no significant differences in the demographics (e.g., size of shelter housing unit, sex, or age distribution) of the vaccinated shelters versus the unvaccinated control shelters included in these studies (table S1). No adverse events were associated with vaccination in this large NHP cohort. To investigate vaccine-mediated immune responses in immunized animals, serum samples were collected just before primary vaccination and at 6 and 12 months after primary vaccination. Serum antibodies were tested against a number of *C. coli* antigens including a total whole-cell lysate and purified flagellin with group geometric mean titers (GMTs) and 95% confidence intervals (CIs) determined at each time point (Fig. 5). Prevaccination RM serum samples demonstrated modest reactivity to *Campylobacter* antigens, as would be anticipated given the persistent environmental exposure. However, H_2_O_2_-Campy_C_ vaccination increased antibody responses above those observed after natural exposure and greatly improved the levels of the total antibacterial antibody response against the whole cell [*P* < 0.001, repeated measures analysis of variance (ANOVA) with Tukey’s multiple test correction; Fig. 5A] and the anti-flagellin antibody response (*P* < 0.001, repeated measures ANOVA with Tukey’s multiple test correction; Fig. 5B). Total *C. coli*–specific antibody responses rose from an initial baseline GMT of 14,031 (95% CI, 11,7191 to 6797) before vaccination to 23,493 (95% CI, 19,008 to 29,035) at 6 months after primary vaccination and reached a titer of 34,888 (95% CI, 28,434 to 42,807) at 12 months (i.e., 6 months after booster vaccination). Serum antibody titers against flagellin showed an even greater increase, starting at a GMT of 18,419 (95% CI, 14,082 to 24,090) at the time of primary vaccination, then increasing to 43,747 (95% CI, 32,466 to 58,949) at 6 months and to 92,042 (95% CI, 76,548 to 110,673) at the 12-month time point. While there was a weak correlation between *C. coli* whole-cell and flagellin-specific antibody titers before vaccination (*R*^2^ = 0.22), these values became increasingly more correlated following primary (*R*^2^ = 0.32) and booster vaccination (*R*^2^ = 0.90) (Fig. 5C). Since purified flagellin is the only antigen on the *y* axis and is a component of the whole-cell *Campylobacter* antigen on the *x* axis, the increased correlations observed in Fig. 5C indicate that the immune response to flagellin becomes more immunodominant in comparison to the other bacterial antigens after each round of vaccination.

To further evaluate the breadth of the antibacterial antibody response, *C. coli* lysate was probed by Western blot with prevaccination and postvaccination immune sera from mice and RM (Fig. 5D). As expected, naïve mouse serum was nonreactive against *C. coli* but showed responses to multiple *Campylobacter* antigens following H_2_O_2_-Campy_C_ immunization. Sera from unvaccinated RM showed weak reactivity against *Campylobacter* antigens by Western blot. However, following H_2_O_2_-Campy_C_ vaccination, additional bands of antibody-reactive antigen became more apparent and appeared to share similarity to the breadth of antibody responses observed in the vaccinated mice (Fig. 5D). While the identity of most of the bacterial antigens has yet to be determined, the protein band migrating at approximately 60 kDa was confirmed to be flagellin based on reactivity with an established flagellin-specific CF5 monoclonal antibody (*13*) and represents an immunodominant antigen following H_2_O_2_-Campy_C_ immunization. In total, these data indicate that *Campylobacter* vaccine–mediated immunity can be induced to a number of bacterial antigens and results in higher and more consistent levels of antibody than that observed after natural infection alone.

### Protection against *Campylobacter*-associated diarrheal disease

Given the high rates of *C. coli*–associated enteric disease among the RM (Fig. 1), we first investigated whether intramuscular (IM) administration of a 40-μg dose of a *C. coli*–based vaccine could provide protective mucosal immunity against clinically defined cases of *C. coli–*associated diarrhea (note that two infants received a 20-μg dose of vaccine instead of the full dose at the discretion of the attending veterinarians). Since most human vaccines require at least two immunizations for optimal protective efficacy and the durability of immunological memory is often improved following booster vaccination, we administered a booster dose 6 months later and then continued to monitor the incidence of diarrheal disease among the vaccinated and unvaccinated NHP. In standard direct-challenge animal models, the infection/exposure to the pathogen of interest is not performed until a sufficient period of time has elapsed following vaccination to allow for an immune response to be mounted. Because exposure to enteric pathogens can occur at any time throughout the year among outdoor-housed RM (Fig. 1), this study was prospectively designed to exclude animals that experienced a diarrheal episode during the first 2 weeks following vaccination, since it is unlikely that this would be enough time for vaccine-mediated immunity to reach a protective threshold (see Materials and Methods). In 2015, the H_2_O_2_-Campy_C_–vaccinated cohort consisted of 60 animals (61 animals minus 1 animal excluded from analysis due to hospitalization 1 day after primary vaccination) including 42 infants/juveniles and 18 adults with a demographic profile that matched the unvaccinated control population (table S1). Disease incidence was monitored for up to 1 year (1 April 2015 to 31 March 2016). Vaccinated animals were followed for an average of 336 days with 49 animals monitored for the entire 365-day observation period, culminating in 20,153 total exposure days in outdoor group housing. During this period of time, a total of two cases of diarrhea were identified among the vaccinated animals and were diagnosed as one case of *C. coli*–associated diarrhea, zero cases of *C. jejuni*–associated diarrhea, and one case of non-*Campylobacter*–associated diarrhea. Unvaccinated contemporaneous control animals (*n* = 1645) were monitored for 269 days on average with 761 animals followed for the entire 365-day observation period, culminating in 442,393 days of exposure. During this period of observation, 141 total cases of diarrhea were recorded among at-risk control animals. The separate *C. coli*–associated diarrheal analysis recorded 125 unique cases of disease, while the *C. jejuni*–associated diarrheal analysis recorded eight cases. Note that there were eight unvaccinated animals housed among the vaccinated NHP cohort in 2015 that either were not available on the day of vaccination or were born after the vaccination date. Of these unvaccinated sentinel animals, two of the eight animals (25%) were hospitalized with *C. coli*–associated diarrhea, indicating that vaccinated animals had continued exposure to *C. coli* during the course of the vaccine trial.

**Fig. 6:**
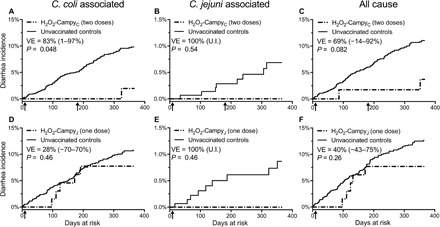
H_2_O_2_-inactivated *Campylobacter* vaccination provides protection against severe cases of diarrheal disease. RMs were immunized with either H_2_O_2_-Campy_C_ (**A** to **C**) or H_2_O_2_-Campy_J_ (**D** to **F**) and monitored for diarrheal episodes, along with unvaccinated control animals in adjacent housing units, for up to 1 year after vaccination. Diarrheal episodes were categorized as *C. coli*–associated diarrhea (A and D), *C. jejuni*–associated diarrhea (B and E), or all-cause diarrhea (C and F). The log-rank test was used to determine *P* values comparing the cumulative risk of diarrhea between vaccinated and unvaccinated groups. Hazard ratios (HRs) and 95% CIs were calculated by inverting the partial-likelihood score test under the Cox proportional hazards model (*40*), with VE defined as 1 − HR × 100%. U.I. indicates undefined CIs. For both vaccine cohort studies, unvaccinated control animals were contemporaneously followed. Arrows represent the dates that vaccinations were performed.

Overall, the incidence rate of *C. coli–*associated diarrhea among the 1645 contemporaneous unvaccinated animals was 9.8% versus 1.96% among the H_2_O_2_-Campy_C_–immunized animals. VE, as determined through the time-to-event Cox proportional hazards model, was estimated at 83% (95% CI, 1 to 97%, *P* = 0.048; Fig. 6A). *C. jejuni*–associated diarrhea cases were rare (0.69% incidence among unvaccinated animals Fig. 6B) and although we found an apparent VE of 100%, this was based on only eight cases identified among the unvaccinated control animals versus zero cases among the 60 H_2_O_2_-Campy_C_–vaccinated animals and was not statistically significant (*P* = 0.54). The incidence of all-cause diarrhea (Fig. 6C) was 11.0% among unvaccinated animals compared to 3.69% among H_2_O_2_-Campy_C_–vaccinated animals, with time-to-event Cox proportional hazard analysis estimating VE of 69% (95% CI, −14 to 92%,) and showed a statistically nonsignificant trend toward reduced enteric disease (*P* = 0.08), likely due to the underlying reduction in *C. coli*–associated enteric disease since it is a major contributor to all-cause diarrhea among these animals (Fig. 1B). To our knowledge, this represents the first demonstration of vaccine-mediated protection against *Campylobacter* using a natural primate model of enteric disease under conditions of repeated exposure through the fecal-oral route of transmission.

Initial results of the H_2_O_2_-Campy_C_ vaccine indicated a trend toward protective immunity during the first 6 months before booster vaccination (*P* = 0.08). On the basis of the positive results observed with the H_2_O_2_-Campy_C_ vaccine, we initiated a pilot study during the following calendar year (2016, table S1) to (i) determine whether a single *Campylobacter* vaccination could elicit protective immunity against diarrheal disease and (ii) test whether a related *C. jejuni*–based vaccine (H_2_O_2_-Campy_J_) would provide cross-protective immunity against *C. coli*. Although the rates of *C. jejuni* in the sheltered-house animals were likely to be too low to assess homologous protection (Figs. 1B and 6, B and E), this approach was designed to determine whether H_2_O_2_-Campy_J_ could provide heterologous vaccine-mediated cross-protection against *C. coli* (Fig. 6D). In 2016, the H_2_O_2_-Campy_J_–vaccinated cohort consisted of 67 animals including 41 infants/juveniles and 26 adults with demographics that matched the unvaccinated control population (see Materials and Methods and table S1). Similar to the 2015 study, disease incidence was monitored for up to 1 year. Vaccinated animals were followed for an average of 343 days with 54 animals monitored for the entire 365-day observation period, culminating in 22,969 total days of exposure. During this period of time, there were five cases of diarrhea that were all diagnosed as *C. coli*–associated with zero cases of *C. jejuni*–associated diarrhea. Unvaccinated contemporaneous control animals (*n* = 1538) were monitored for an average of 239 days with 759 animals followed for the entire 365-day observation period, culminating in 367,792 days of exposure. During this period of observation, there were 138 total cases of diarrhea recorded including 115 cases of *C. coli*–associated diarrhea and 9 cases of *C. jejuni*–associated diarrhea. There were 19 unvaccinated sentinel animals that were housed among the 67 H_2_O_2_-Campy_J_–immunized cohort, and 1 of the 19 (5.3%) animals was diagnosed with *C. coli*–associated diarrhea. During the 1-year period following vaccination, the *C. coli*–associated diarrhea incidence reached 10.8% in the unvaccinated contemporaneous controls versus a rate of 7.8% in the H_2_O_2_-Campy_J_ vaccine recipients (Cox proportional hazard VE, 28%; 95% CI, −70 to 70%, *P* = 0.46). Similar to the *C. coli*–based vaccine studies performed in 2015, in these 2016 vaccine studies, there were nine cases of *C. jejuni*–associated diarrhea among the unvaccinated controls (0.87% incidence rate) with no *C. jejuni* cases observed among the 67 H_2_O_2_-Campy_J_–vaccinated animals that were monitored for diarrheal illness for up to 1 year after vaccination (0% incidence of *C. jejuni*–associated diarrhea, *P* = 0.46). When comparing all-cause diarrhea, the incidence density among the vaccinated animals reached 7.7%, compared to a rate of 12.6% among controls (Cox proportional hazard VE, 40%; 95% CI, −43 to 75%). Although VE against all-cause diarrhea did not reach statistical significance (*P* = 0.26), it showed a trend that is consistent with the results observed after H_2_O_2_-Campy_C_ vaccination.

Analysis of the published genome for the *C. jejuni* strain used in the H_2_O_2_-Campy_J_ vaccine [strain CG8421 (*6*)] indicated that similar to the H_2_O_2_-Campy_C_ vaccine, it did not match the CPS and LOS loci relative to the *C. coli* strains that circulated in the NHP colony in 2015 or 2016 (Fig. 2 and Fig. 3). However, the major flagellin gene products (flagellins A and B) demonstrated 89 to 95% sequence identity to the primary *C. coli* isolates circulating between 2015 and 2016 (table S2) and suggest that flagellin and/or other conserved surface proteins may provide a mechanism to explain potential cross-protective immunity. In total, these studies show statistically significant vaccine-mediated protection against *C. coli*–associated disease with the potential for at least partial cross-species protection against heterologous species of *Campylobacter* that should be further explored.

## DISCUSSION

Campylobacteriosis is one of the most common bacterial infections worldwide, with an annual estimated burden of >25,000 deaths in children under 5 years of age (*14*). In addition to acute gastrointestinal symptoms, recent studies have revealed that infection with *Campylobacter* is associated with poor growth outcomes for children in low-income settings (*3*). In the studies presented here, we used a natural acquisition model of *C. coli and C. jejuni* infection among RM to test novel H_2_O_2_-inactivated *Campylobacter* vaccines. Our experimental *Campylobacter* vaccine provided up to 83% VE against *C. coli*–associated diarrheal disease. These results indicate that an H_2_O_2_-inactivated *Campylobacter* vaccine may be a viable option toward the development of a human vaccine against this important enteric pathogen.

The NHP model of *Campylobacter*-associated disease described here is unique in the sense that it relies on natural exposure and fecal-oral routes of transmission. This model may also be useful for determining potential immune correlates that are associated with protection against *Campylobacter*-associated diarrhea. In our experiments, we had only a single breakthrough case *C. coli*–associated diarrhea after *C. coli* vaccination, and so the sample size was too small to determine correlates of immunity. Nevertheless, future studies, preferably using graded doses of vaccine to elicit varying levels of vaccine-mediated immunity, could be useful for identifying an immune correlate against this important diarrheal disease. Poultry represents another commonly used model of *Campylobacter* infection, but *C. jejuni* is often considered a commensal organism that generally does not cause overt disease (*15*). Direct gastric challenge models using ferrets have been reported, although the need for compounds that inhibit peristalsis, the use of high challenge doses [10^10^ to 10^11^ colony-forming unit (CFU)] and the relatively mild *Campylobacter*-associated disease, limits the relevance of this approach (*16*). Oral challenge studies in rats have demonstrated colonization but reported a lack of clinical symptoms (*17*). A more recent report used high-dose oral challenge in rats to induce an irritable bowel syndrome–like disease (*18*), but infections were cleared rapidly (≤14 days) and no animals had watery stool, which is inconsistent with human disease. Gnotobiotic piglets are susceptible to *Campylobacter* infection, whereas standard-housed piglets are generally resistant to overt disease (*16*). Similarly, both oral and intraperitoneal *Campylobacter* challenges in laboratory mice typically result in subclinical infection (*16*) with the exception of the recently developed antibiotic pretreatment model in zinc-deficient animals that show several characteristics of human campylobacteriosis after oral challenge (*19*). Infection of MyD88 knockout mice, in conjunction with vancomycin antibiotic treatment, has shown the ability to induce colonic pathology but failed to provoke overt diarrhea (*20*). Rabbit models require surgery and direct application of bacteria to the intestinal tract, but the relevance to human disease is uncertain due to the artificial nature of the infection and the trauma associated with the surgery (*16*). Direct intragastric challenge of RMs with *C. jejuni* may induce clinical signs of diarrhea (*5*), but results have been variable and require doses as high as 10^11^ CFU to elicit disease. Even under these conditions, about one in five animals may fail to show any signs of diarrhea (*5*). The New World monkey, *Aotus nancymaae*, has also been proposed as an alternative NHP challenge model but requires doses as high as 5 × 10^12^ CFU of *C. jejuni* to induce relatively mild illness in ~80% of animals (*21*). The limitations observed with direct challenge studies in NHP have also been encountered in human challenge studies. A meta-analysis of direct human and NHP challenge studies demonstrated that both species require similarly high doses of *C. jejuni* to cause overt disease, with doses that are 100-fold to 10 million–fold higher than those found during natural infection (*22*). While the reason for these discrepancies is unclear, these results suggest that direct gastric challenge of primates with laboratory-cultured *Campylobacter* may not be optimal compared to a natural exposure model for pathogenesis and vaccine development studies.

Most small laboratory animals are maintained under specific pathogen–free conditions, whereas the RM model provides the opportunity to study more complex enteric disease under conditions that involve coinfections more similar to those observed in humans living in endemic low- and middle-income countries. Likewise, our NHP colony is endemic for several human enteric pathogens including *Campylobacter*, *Shigella flexneri*, *Shigella dysenteriae*, *Cryptosporidium*, and *Giardia* among other bacterial and parasitic pathogens. A recent study also found the RM gut microbiome was more closely related to established microbiome datasets for Malawian and Amerindian populations than to an American microbiome (*23*). We believe that development of a *Campylobacter* vaccine that is effective under these conditions of comorbidity is more likely to be successful in field trials than vaccines against *Campylobacter* that are studied only in isolation. Although the correlates of immunity for *Campylobacter* remain largely undefined, T cells are unlikely to play a direct role in protection since these are extracellular bacterium. Studies in several animal models (*24*, *25*) and studies of naturally infected humans (*26*, *27*) have demonstrated that flagellin is a dominant target of the humoral immune response. Although results from an initial phase 1 human clinical trial involving intranasal vaccination with a subunit flagellin-based vaccine were suboptimal (*28*), in poultry vaccination trials, flagellin-specific antibody responses have been associated with a 100- to 1000-fold reduction in *C. jejuni* cecal colonization (*29*), underscoring the potential importance of mounting strong antibody responses to this and possibly other bacterial surface antigens. A recombinant flagellin subunit vaccine was also able to demonstrate heterologous protection in a mouse intranasal challenge model of *C. jejuni* (*30*). In our studies, vaccination of RM with a whole-cell vaccine resulted in *Campylobacter*-specific antibody levels that were higher than that observed following natural exposure and greatly enhanced the levels of anti-flagellin antibodies (Fig. 5). It is possible that the high immunogenicity of flagellin in our vaccine formulation is due, at least in part, to maintaining conformational and structural integrity of the antigen on the intact bacterial cell surface in a manner that is more similar to the antigen presentation that would occur during active infection. The vaccine-mediated antibody responses to flagellin observed in these NHP appears to be analogous to earlier studies of naturally infected humans which indicated that “This protein has a particularly high ratio of antigenicity to molar representation on the outer membrane.” (*27*). The protection data against circulating bacterial strains that differed at the LOS and CPS loci (Figs. 2 and 3) also suggest that these elevated antibody responses to the more highly conserved flagellin protein, and potentially other conserved surface proteins (*28*), may be important in vaccine-mediated control of *Campylobacter*-associated enteric disease.

Although *Campylobacter* is a mucosal pathogen, mucosal vaccination was not required to induce protective immunity. Similarly, outer membrane vesicle (OMV) vaccines against *Neisseria meningitidis*, a Gram-negative bacterium, are also administered intramuscularly and have demonstrated up to 80 to 85% efficacy against invasive forms of disease (*31*). Besides providing broad immunity at the species level, the *N. meningitidis* vaccine approach also appears to hold promise even at the genus level. In a recent retrospective case-control study in New Zealand, an OMV-based *N. meningitidis* vaccine showed 31% efficacy (95% CI, 21 to 39%) against *Neisseria gonorrhoeae* (*32*). While this level of protection was modest, this result suggests that vaccines that contain multiple bacterial surface antigens provide broad immune responses and enhanced protective immunity, even against more distantly related mucosal bacteria. For the *Campylobacter* vaccine approach presented here, it is notable that the *C. coli* and *C. jejuni* vaccine strains did not match the LOS or CPS of the bacterial strains isolated during 2015 and 2016, the years in which vaccine field trials were conducted (Figs. 2 and 3). The central domains of the *Campylobacter* flagellin proteins can vary across strains and species, but the N and C termini maintain a high level of amino acid homology (*33*) and this sequence homology (or perhaps similarity among other surface proteins) may have contributed to the potential heterologous cross-protection against *C. coli* observed using the H_2_O_2_-Campy_J_ vaccine, similar to the results observed for *N. meningitidis* vaccine–mediated protection against *N. gonorrhoeae* (*32*).

Although the natural exposure model of *Campylobacter*-associated diarrhea among outdoor-housed RM has advantages over other animal models, it also has several limitations. Unlike experimental direct-challenge models used for other pathogens, this natural exposure model does not have 100% disease penetrance. With annual *C. coli*–associated diarrhea incidence of 9.8 to 10.8% during the 2015 and 2016 trials, respectively, a relatively large sample size is needed to obtain statistically significant data. A single breakthrough case of *C. coli*–associated diarrhea among the 60 H_2_O_2_-Campy_C_–immunized animals reduced VE from 100 to 83% VE when compared to the rates of disease observed among the unvaccinated control animals. Likewise, despite achieving what appeared to be 100% VE against *C. jejuni*–associated diarrheal disease, the incidence rate among the controls (0.69 and 0.83% disease incidence in 2015 and 2016, respectively) precluded definitive statistical analysis. Another challenge to this natural exposure model is that animals are at risk of enteric infection during and shortly after vaccination before a protective immune response can be mounted. We anticipated this issue and designed the experimental approach to exclude diarrheal animals that were diagnosed during the first 2 weeks after primary vaccination of our two-dose vaccination series. This resulted in the exclusion of one animal from the H_2_O_2_-Campy_C_–immunized cohort due to diarrhea-associated hospitalization within 1 day after vaccination. This animal was likely beginning to be symptomatic before vaccination but reached the threshold for requiring hospitalization based on degree of dehydration, lethargy, and body score, as well as loose stool (*8*). If it was included in the experimental analysis, then the apparent VE would be 65% (*P* = 0.12). However, this is not an appropriate comparison because this current study focused on the development of a preventative vaccine against *Campylobacter*-associated diarrhea and is not designed to function as a therapeutic vaccine and is therefore unlikely to provide protection against disease once clinical manifestations of the enteric infection have already begun. Further studies to refine preclinical NHP models should be explored and eventual development of an optimized direct-challenge approach will greatly facilitate future testing of safe and effective vaccines against *Campylobacter*-associated disease.

To date, only a limited number of *Campylobacter* vaccine strategies have been examined in humans. Preliminary studies with a formalin-inactivated vaccine were conducted using an oral vaccination route and showed no protection and signs of diarrheal disease after ingestion of high vaccine doses (*28*). An intranasal vaccine approach was attempted for a recombinant flagellin candidate, but the vaccine has not advanced due to poor immunogenicity (*28*). ACE393 is a more recent *Campylobacter* vaccine candidate focused on a single protein identified through proteomic screens (*34*). This vaccine was administered as an IM immunization but provided no clinical protection following controlled live challenge in humans (*28*). Currently, a CPS vaccine candidate represents the most advanced *C. jejuni* vaccine approach being pursued in the clinical setting (*10*). In studies performed in *A. nancymaae* monkeys, a three-dose subcutaneous regimen was able to protect against homologous gastric challenge (*35*). As noted in the study, it is unlikely that a single defined CPS will be protective against a large range of *C. jejuni* strains (*10*), and this approach may require multiple CPS antigens for broad vaccine-mediated protection. Multiple serotypes for *C. coli* and *C. jejuni* have been identified on the basis of two serotyping protocols, Penner and Lior. Both serotyping approaches were developed as cataloging methods to study the epidemiology of *Campylobacter* outbreaks before the implementation of modern genetic tools, and their importance in relation to cross-protection and VE remain unclear. In the absence of specific *Campylobacter* strain pre-adsorption, reference sera demonstrate substantial cross-reactivity, suggesting that the whole-cell immunization approach used to develop these reference sera actually elicits a relatively broad response to a number of different serotypes. For instance, in a ferret model of *C. jejuni* disease, heterologous cross-protection was observed between different Lior strains, ranging from 67 to 84% VE against overt signs of diarrhea (*36*). Other studies in small-animal models have likewise demonstrated heterologous protection to different *Campylobacter* serotypes using bacterial whole-cell approaches (*37*). Successful whole-cell inactivated *Campylobacter* vaccines against *C. jejuni* and *Campylobacter fetus fetus* have been used commercially in sheep vaccines for many years (e.g., Campylobacter Fetus–Jejuni Bacterin and CampyVax). Campylovexin was developed to prevent *Campylobacter*-associated abortions in sheep, with ~80% efficacy, and studies indicate that it is protective against 26 different circulating strains of *C. fetus fetus* (Campylovexin Product Profile, Virbac Animal Health, New Zealand, 2015). Together, these reports support our *Campylobacter* vaccine approach, which uses an advanced whole-cell formulation developed to preserve antigenic structures and achieve antibacterial antibody titers that exceed those observed following natural exposure.

In this study, we demonstrate significant VE against naturally occurring *Campylobacter* infection using a novel peroxide-inactivated whole-cell vaccine. Vaccine-mediated protection reached 83% in our field trials despite the vaccine strain expressing CPS and LOS that were genetically mismatched from the circulating strains of *Campylobacter*. Regardless of the *Campylobacter* vaccine strain(s) used for future clinical development, our results demonstrate that significant vaccine-mediated protection against *Campylobacter*-associated diarrhea is feasible and supports the continued development of new and advanced vaccines against these globally relevant enteric pathogens.

## MATERIALS AND METHODS

### Vaccine production

Production lots of *C. coli* (NTICC13) or *C. jejuni* [CG8421 strain provided by P. Guerry at the Naval Medical Research Center (*6*)] were grown to late-log phase in shaker flasks of supplemented growth medium and concentrated/purified by tangential flow filtration. Inactivation was initiated with the addition of 0.10% H_2_O_2_ and 2 μM CuCl_2_, with a total inactivation time of 20 to 22 hours at room temperature (RT). Inactivation was stopped by the addition of excess EDTA and catalase before being adsorbed to 0.10% alum. Five percent of the bulk material for each lot was tested for residual live bacteria under both microaerophilic and standard atmosphere conditions, for a total testing volume of 10%. Each lot was tested for LOS content using an end point chromogenic limulus amebocyte lysate assay (Lonza), with an average value of 9000 endotoxin units (EU) ± 3598 (±SEM) per 40-μg dose determined among four production lots. While there is no precise relationship between EU and endotoxin mass, a range of 5 to 10 EU/ng has been reported for established endotoxin standards (*38*). Using an average value of 7.5 EU/ng, we estimate ~1.2 μg of bacterial LOS per dose, which is similar to the vaccine against *N. meningitidis* (BEXSERO) with a range of 1 to 3 μg of LOS per dose (*39*).

### Campylobacter WGS and phylogenetic analysis

*Campylobacter* fecal isolates were collected over a 3-year period from a sampling of healthy asymptomatic RM carriers and animals hospitalized with *Campylobacter*-associated diarrhea. All isolates were further streaked for purity before a final expansion and DNA extractions. Genomic DNA libraries were generated using iGenomX Riptide (Carlsbad, CA) high throughput rapid library prep following the manufacturer’s suggested protocol using 50 ng of input DNA per sample. Each library was prepared using a unique molecular identifier. The resulting multiplexed library was sequenced on an Illumina MiSeq (San Diego, CA) using MiSeq v2 reagents paired end 2 × 150–base pair reads. Reads were demultiplexed using fulcrum genomics DemuxFastqs tool (--read-structures = 8B12M130T 8M142T --max-mismatches = 0). Trimmed, demultiplexed reads were assembled into contigs using SPAdes genome assembler (cab.spbu.ru/software/spades/; default settings) and submitted to the Pathosystems Resource Integration Center (PATRIC; www.patricbrc.org) for annotation via the RAST toolkit (rast.theseed.org). Reference and assembled genomes were placed in a phylogenetic tree using PATRICs’ Phylogenetic Tree Building service with a final alignment via FastTree (microbesonline.org/fasttree/) using no automated progressive refinement (fig. S2). To allow for expanded flagellin gene sequence analysis, a subset of samples was also sequenced using the long-read PacBio Sequel platform (GENEWIZ, South Plainfield, NJ).

### Campylobacter antigen ELISAs

For *C. coli* specific enzyme-linked immunosorbent assays (ELISAs), whole-cell lysate, purified flagellin, and purified LOS were produced. For whole-cell lysate, bacteria were grown in suspension and inactivated using the optimized H_2_O_2_-based approach as described above. Samples were then sonicated, aliquoted, and held at ≤−65°C. To produce purified flagellin, *C. coli* was grown and concentrated by tangential flow filtration, resuspended in phosphate-buffered saline (PBS), and homogenized by sonication. Homogenates were centrifuged at 2000*g* for 30 min and resuspended in PBS, followed by ultracentrifugation for 3 hours at 100,000*g*. Purified flagellin pellets were resuspended in PBS, aliquoted, and stored at ≤−65°C. Silver-stained SDS–polyacrylamide gel electrophoresis gels run under reduced conditions showed a major band migrating at ~60 kDa that reacted with the flagellin-specific CF5 monoclonal antibody (*13*). For ELISA tests, serum samples were first preadsorbed to reduce nonspecific binding by diluting serum 1:100 into suspensions of heat-killed (56°C for 1 hour) *S. flexneri* (containing approximately 10^9^ CFU/ml) for 60 min at RT, followed by brief centrifugation to collect the preadsorbed serum samples.

Serum ELISA was performed as described in (*9*). Briefly, ELISA plates were coated overnight at 2° to 8°C using optimal concentrations of each antigen as determined through small-scale pilot studies. Unbound antigen was removed, and plates were treated with blocking buffer [5% nonfat dry milk in PBS-T (PBS supplemented with 0.05% Tween 20)] for 1 hour at RT. Plates were rinsed one time with PBS-T and incubated for 1 hour with serial dilutions of the adsorbed serum samples. Plates were washed five times with PBS-T and incubated with an optimal dilution of a goat anti-monkey immunoglobulin Gγ–horseradish peroxidase antibody (Rockland, Limerick, PA) for 1 hour at RT. After a final wash, plates were developed with *o*-phenylenediamine dihydrochloride substrate for 20 min, with development stopped by the addition of an equal volume of 1 M HCl. Optical densities (ODs) were measured at 490 nm, and a log-log transformation of the OD versus reciprocal serum dilution was performed. End point titers were determined as the reciprocal of the serum dilution needed to reach an OD of 0.10. Each plate contained a serum standard to allow normalization between experiments, and each sample was tested in duplicate, with the average value between duplicates taken as the final titer.

### Safety assessment in mice and RMs

For preliminary safety studies, 8-week-old female BALB/c mice were obtained from the Jackson laboratory (Bar Harbor, ME) and immunized by the intraperitoneal route with a 40-μg dose of H_2_O_2_-Campy_C_. In a separate control study, mice were treated with 10 μg of *E. coli* LPS (O111:B4, List Biological Laboratories, Campbell, CA). Animal weights were followed for up to 1 week after immunization. The second phase of our safety assessment was conducted in RM. Two adult females received an IM vaccination with the H_2_O_2_-Campy_C_ vaccine, and two adult females received a mock, alum-only IM vaccination. Health metrics, including weight, attitude, appetite, urine, stool, temperature, and vaccine site, were monitored daily for 14 days.

### Immunization of mice and RMs

To produce *Campylobacter* hyper-immune sera for Western blot staining, mice were immunized with 40 μg of H_2_O_2_-Campy_C_ at 0, 2, and 3 months. Serum samples were collected at 1 month following the final immunization. The ONPRC maintains an active RM breeding colony, and for the H_2_O_2_-Campy_C_ vaccine study, animals were vaccinated intramuscularly with alum-adjuvanted, inactivated *C. coli* vaccine in March 2015 (table S1). At the discretion of the attending veterinarian, two infants received a 0.5-ml (20 μg) primary dose, with all other animals receiving a 1.0-ml (40 μg) primary dose. Animals that were hospitalized with diarrhea during the first 14 days after the first vaccination were a priori excluded from analysis, since these animals are housed in a high exposure setting and 14 days is unlikely to be enough time for the vaccine to elicit a protective immune response. Among the first RM cohort, one adult female experienced a diarrheal episode 1 day after H_2_O_2_-Campy_C_ vaccination, leaving a total of 60 immunized animals that were monitored for diarrheal disease for up to 1 year beginning on 1 April, 2015. Of these animals, 53 were available to receive a 40-μg 6-month booster vaccination, and 49 had matched serum samples available at all time points for serological analysis of antibacterial immunity. In July 2016, a second cohort of RM was vaccinated with a single IM 40-μg dose of the alum-adjuvanted *C. jejuni* vaccine, H_2_O_2_-Campy_J_ (table S1). Three animals experienced diarrhea within 2 weeks of primary vaccination, resulting in a final cohort of 67 animals that were monitored for diarrheal disease for up to 1 year beginning on 3 August 2016.

### Diarrheal disease incidence

Diarrhea incidence rates among vaccinated animals were tracked for a 1-year period after primary vaccination using a centralized electronic health record system (*7*) and compared contemporaneously to unvaccinated control animals during that same year of disease activity. Any animals exhibiting signs of severe diarrheal illness were transported to the veterinary hospital for further evaluation. If a case of diarrhea was confirmed, then rectal fecal cultures were submitted to the ONPRC Clinical Pathology Laboratory for *C. coli*, *C. jejuni*, and *Shigella* spp. diagnosis and the results were documented in a searchable central database (PRIMe). RMs were observed daily for the entire year by trained husbandry staff, including personnel who were blinded to the animal’s vaccination history. For all-cause diarrhea analysis, animals were considered to have met the end point with their first recorded case of any diarrheal episode. For *C. coli*–associated diarrhea, animals met the end point with their first recorded case of *C. coli*–associated diarrhea, regardless of whether they were previously diagnosed with *C. jejuni*–associated diarrhea or with unrelated/all-cause diarrhea. For *C. jejuni*–associated diarrhea, animals met the end point with their first recorded case of *C. jejuni*–associated diarrhea, regardless of whether they were previously diagnosed with *C. coli*–associated diarrhea or with unrelated/all-cause diarrhea. *C. coli*– and *C. jejuni*–associated diarrhea diagnoses were performed by blinded personnel in the ONPRC Clinical Pathology Laboratory who were not aware of the animal’s vaccination history.

### Statistics

Figures show group averages ± SEM. Correlation between *Campylobacter* whole-cell lysate and flagellin ELISA titers were determined by linear regression following logarithmic transformation. Statistical comparisons of antibacterial antibody levels were made on logarithm transformed titers using repeated measures ANOVA with Tukey’s multiple test correction. In the experimental design stage, power analysis was performed to estimate necessary cohort sizes using Fisher’s two-tailed exact test (G*Power, version 3.1.9.2, Heinrich-Heine-Universität Düsseldorf). An annual *C. coli*–associated diarrhea rate of 13% was assumed for unvaccinated animals, with an 18:1 allocation of unvaccinated to vaccinated animals. Using these parameters, we estimated that ≥50 vaccinated animals, compared to at least 900 unvaccinated animals, would provide ≥80% power to detect an effect size of 0.10 at the 0.05 significance level. Similar to human clinical trials/field studies, any animal removed from the study for unrelated reasons was considered lost to follow-up, but their data were included in the final dataset up to the date of removal. Given that each shelter unit typically houses 30 to 40 animals, we chose to vaccinate two shelters and compare diarrheal incidence rates relative to a small number of unvaccinated animals within the two shelters (*n* = 8 unvaccinated animals in 2015 and *n* = 19 unvaccinated animals in 2016 were housed with the vaccinated cohorts), together with the >1500 unvaccinated control animals housed in adjacent shelters (table S1). The unvaccinated sentinel animals that were housed among the vaccinated cohorts were not by design but occurred because the animals were either temporarily located elsewhere on the day of vaccination or were born after the vaccination date. For both vaccine cohorts, all animals were considered at risk while located in shelter housing during each 1-year period following vaccination. Ideally, statistical modeling for VE should account for animal clustering. To examine the effect of reduction in effective sample size, we performed a mixed effects Cox regression model to account for the correlation between outcome measurements collected for monkeys from the same shelter. This approach yielded a marginally nonsignificant result: H_2_O_2_-Campy_C_ vaccination provided 87% VE (95% CI, −18 to 99%, *P* = 0.07) against *C. coli*–associated diarrhea. However, this approach is problematic due to husbandry and logistical needs among the breeding groups that resulted in animals being moved from one shelter to another or being moved from one breeding group to another, throughout the course of the year. As a result, shelter membership may not be a proper representation of clustering structure. Therefore, for disease acquisition studies in Fig. 6, the log-rank test was used for comparing the cumulative risk of diarrhea between vaccinated and unvaccinated groups. In addition, VE was based on time to disease and defined as [1 – hazard ratio (HR)] × 100%. The HR and associated 95% CIs were obtained by inverting the partial-likelihood score test under the Cox proportional hazards model as described in (*40*). Statistical analyses were performed using R3.6.0 (R Foundation for Statistical Computing, Vienna, Austria, 2019) and SAS9.4 (SAS Institute, Cary, NC).

### Study approval

All animal studies were overseen and approved by the Oregon Health and Science University Institutional Animal Care and Use Committee in accordance with the National Institutes of Health guide for the care and use of laboratory animals. Animals were housed in accordance with standards established by the U.S. Federal Animal Welfare Act and the *Guide for the Care and Use of Laboratory Animals*.

## Supplementary Material

aba4511_SM.pdf

## SUPPLEMENTARY MATERIALS

Supplementary material for this article is available at http://advances.sciencemag.org/cgi/content/full/6/26/eaba4511/DC1

## Appendix

View/request a protocol for this paper from *Bio-protocol*.

## References

[1] Kotloff K. L., Nataro J. P., Blackwelder W. C., Nasrin D., Farag T. H., Panchalingam S., Wu Y., Sow S. O., Sur D., Breiman R. F., Faruque A. S., Zaidi A. K., Saha D., Alonso P. L., Tamboura B., Sanogo D., Onwuchekwa U., Manna B., Ramamurthy T., Kanungo S., Ochieng J. B., Omore R., Oundo J. O., Hossain A., Das S. K., Ahmed S., Qureshi S., Quadri F., Adegbola R. A., Antonio M., Hossain M. J., Akinsola A., Mandomando I., Nhampossa T., Acacio S., Biswas K., O'Reilly C. E., Mintz E. D., Berkeley L. Y., Muhsen K., Sommerfelt H., Robins-Browne R. M., Levine M. M., Burden and aetiology of diarrhoeal disease in infants and young children in developing countries (the Global Enteric Multicenter Study, GEMS): A prospective, case-control study. Lancet 382, 209–222 (2013). 23680352 10.1016/S0140-6736(13)60844-2

[2] J. C. Buzby, T. Roberts, B. M. Allos, Estimated annual costs of *Campylobacter*-associated Guillain-Barré syndrome (USDA Economic Research Service Agricultural Economic Report July, 1997) pp. 1–33.

[3] Lee G., Pan W., Peñataro Yori P., Paredes Olortegui M., Tilley D., Gregory M., Oberhelman R., Burga R., Chavez C. B., Kosek M., Symptomatic and asymptomatic *Campylobacter* infections associated with reduced growth in Peruvian children. PLOS Negl. Trop. Dis. 7, e2036 (2013). 23383356 10.1371/journal.pntd.0002036 PMC3561130

[4] Sainato R., ElGendy A., Poly F., Kuroiwa J., Guerry P., Riddle M. S., Porter C. K., Epidemiology of *Campylobacter* infections among children in Egypt. Am. J. Trop. Med. Hyg. 98, 581–585 (2018). 29260646 10.4269/ajtmh.17-0469 PMC5929192

[5] Islam D., Lewis M. D., Srijan A., Bodhidatta L., Aksomboon A., Gettayacamin M., Baqar S., Scott D., Mason C. J., Establishment of a non-human primate *Campylobacter* disease model for the pre-clinical evaluation of *Campylobacter* vaccine formulations. Vaccine 24, 3762–3771 (2006). 16098634 10.1016/j.vaccine.2005.07.023

[6] Poly F., Read T. D., Chen Y. H., Monteiro M. A., Serichantalergs O., Pootong P., Bodhidatta L., Mason C. J., Rockabrand D., Baqar S., Porter C. K., Tribble D., Darsley M., Guerry P., Characterization of two *Campylobacter jejuni* strains for use in volunteer experimental-infection studies. Infect. Immun. 76, 5655–5667 (2008). 18809665 10.1128/IAI.00780-08 PMC2583572

[7] Prongay K., Park B., Murphy S. J., Risk factor analysis may provide clues to diarrhea prevention in outdoor-housed rhesus macaques (Macaca mulatta). Am. J. Primatol. 75, 872–882 (2013). 23568382 10.1002/ajp.22150 PMC3956043

[8] Haertel A. J., Prongay K., Gao L., Gottlieb D. H., Park B., Standard growth and diarrhea-associated growth faltering in captive infant rhesus macaques (Macaca mulatta). Am. J. Primatol. 80, e22923 (2018). 30281825 10.1002/ajp.22923 PMC6405262

[9] Amanna I. J., Raué H. P., Slifka M. K., Development of a novel hydrogen peroxide-based vaccine platform. Nat. Med. 18, 974–979 (2012). 22635006 10.1038/nm.2763 PMC3506259

[10] Maue A. C., Poly F., Guerry P., A capsule conjugate vaccine approach to prevent diarrheal disease caused by *Campylobacter jejuni*. Hum. Vaccin. Immunother. 10, 1499–1504 (2014). 24632556 10.4161/hv.27985 PMC5396224

[11] Russell R. G., Sarmiento J. I., Fox J., Panigrahi P., Evidence of reinfection with multiple strains of *Campylobacter jejuni* and *Campylobacter coli* in Macaca nemestrina housed under hyperendemic conditions. Infect. Immun. 58, 2149–2155 (1990). 2365455 10.1128/iai.58.7.2149-2155.1990 PMC258790

[12] Lastovica A. J., Le Roux E., Congi R. V., Penner J. L., Distribution of sero-biotypes of *Campylobacter jejuni* and *C. coli* isolated from paediatric patients. J. Med. Microbiol. 21, 1–5 (1986). 3950960 10.1099/00222615-21-1-1

[13] Nuijten P. J., van der Zeijst B. A., Newell D. G., Localization of immunogenic regions on the flagellin proteins of *Campylobacter jejuni* 81116. Infect. Immun. 59, 1100–1105 (1991). 1705240 10.1128/iai.59.3.1100-1105.1991 PMC258373

[14] Poly F., Noll A. J., Riddle M. S., Porter C. K., Update on *Campylobacter* vaccine development. Hum. Vaccin. Immunother. 15, 1389–1400 (2019). 30252591 10.1080/21645515.2018.1528410 PMC6663129

[15] Hermans D., Van Deun K., Martel A., Van Immerseel F., Messens W., Heyndrickx M., Haesebrouck F., Pasmans F., Colonization factors of *Campylobacter jejuni* in the chicken gut. Vet. Res. 42, 82 (2011). 21714866 10.1186/1297-9716-42-82 PMC3156733

[16] Newell D. G., Animal models of *Campylobacter jejuni* colonization and disease and the lessons to be learned from similar Helicobacter pylori models. Symp. Ser. Soc. Appl. Microbiol. 90, 57S–67S (2001). 10.1046/j.1365-2672.2001.01354.x 11422561

[17] Epoke J., Coker A. O., Intestinal colonization of rats following experimental infection with *Campylobacter jejuni*. East Afr. Med. J. 68, 348–351 (1991). 1935728

[18] Pimentel M., Chatterjee S., Chang C., Low K., Song Y., Liu C., Morales W., Ali L., Lezcano S., Conklin J., Finegold S., A new rat model links two contemporary theories in irritable bowel syndrome. Dig. Dis. Sci. 53, 982–989 (2008). 17934822 10.1007/s10620-007-9977-z

[19] Giallourou N., Medlock G. L., Bolick D. T., Medeiros P. H., Ledwaba S. E., Kolling G. L., Tung K., Guerry P., Swann J. R., Guerrant R. L., A novel mouse model of *Campylobacter jejuni* enteropathy and diarrhea. PLOS Pathog. 14, e1007083 (2018). 29791507 10.1371/journal.ppat.1007083 PMC5988333

[20] Stahl M., Ries J., Vermeulen J., Yang H., Sham H. P., Crowley S. M., Badayeva Y., Turvey S. E., Gaynor E. C., Li X., Vallance B. A., A novel mouse model of *Campylobacter jejuni* gastroenteritis reveals key pro-inflammatory and tissue protective roles for Toll-like receptor signaling during infection. PLOS Pathog. 10, e1004264 (2014). 25033044 10.1371/journal.ppat.1004264 PMC4102570

[21] Jones F. R., Baqar S., Gozalo A., Nunez G., Espinoza N., Reyes S. M., Salazar M., Meza R., Porter C. K., Walz S. E., New World monkey Aotus nancymae as a model for *Campylobacter jejuni* infection and immunity. Infect. Immun. 74, 790–793 (2006). 16369042 10.1128/IAI.74.1.790-793.2006 PMC1346678

[22] Teunis P. F. M., Bonačić Marinovic A., Tribble D. R., Porter C. K., Swart A., Acute illness from *Campylobacter jejuni* may require high doses while infection occurs at low doses. Epidemics 24, 1–20 (2018). 29456072 10.1016/j.epidem.2018.02.001

[23] Yasuda K., Oh K., Ren B., Tickle T. L., Franzosa E. A., Wachtman L. M., Miller A. D., Westmoreland S. V., Mansfield K. G., Vallender E. J., Miller G. M., Rowlett J. K., Gevers D., Huttenhower C., Morgan X. C., Biogeography of the intestinal mucosal and lumenal microbiome in the rhesus macaque. Cell Host Microbe 17, 385–391 (2015). 25732063 10.1016/j.chom.2015.01.015 PMC4369771

[24] Burr D. H., Caldwell M. B., Bourgeois A. L., Morgan H. R., Wistar R. Jr., Walker R. I., Mucosal and systemic immunity to *Campylobacter jejuni* in rabbits after gastric inoculation. Infect. Immun. 56, 99–105 (1988). 3335413 10.1128/iai.56.1.99-105.1988 PMC259241

[25] Cawthraw S., Ayling R., Nuijten P., Wassenaar T., Newell D. G., Isotype, specificity, and kinetics of systemic and mucosal antibodies to *Campylobacter jejuni* antigens, including flagellin, during experimental oral infections of chickens. Avian Dis. 38, 341–349 (1994). 7526839

[26] Nachamkin I., Hart A. M., Western blot analysis of the human antibody response to *Campylobacter jejuni* cellular antigens during gastrointestinal infection. J. Clin. Microbiol. 21, 33–38 (1985). 2578479 10.1128/jcm.21.1.33-38.1985 PMC271575

[27] Wenman W. M., Chai J., Louie T. J., Goudreau C., Lior H., Newell D. G., Pearson A. D., Taylor D. E., Antigenic analysis of *Campylobacter* flagellar protein and other proteins. J. Clin. Microbiol. 21, 108–112 (1985). 2578478 10.1128/jcm.21.1.108-112.1985 PMC271585

[28] D. Tribble, S. Baqar, S. A. Thompson, in *Development of a Human Vaccine: Campylobacter (Third Edition)*, I. Nachamkin, C. M. Szymanski, M. J. Blaser, Eds. (ASM Press, 2008), pp. 429–444.

[29] Neal-McKinney J. M., Samuelson D. R., Eucker T. P., Nissen M. S., Crespo R., Konkel M. E., Reducing *Campylobacter jejuni* colonization of poultry via vaccination. PLOS ONE 9, e114254 (2014). 25474206 10.1371/journal.pone.0114254 PMC4256221

[30] Lee L. H., Burg E. III, Baqar S., Bourgeois A. L., Burr D. H., Ewing C. P., Trust T. J., Guerry P., Evaluation of a truncated recombinant flagellin subunit vaccine against *Campylobacter jejuni*. Infect. Immun. 67, 5799–5805 (1999). 10531231 10.1128/iai.67.11.5799-5805.1999 PMC96957

[31] Galloway Y., Stehr-Green P., McNicholas A., O'Hallahan J., Use of an observational cohort study to estimate the effectiveness of the New Zealand group B meningococcal vaccine in children aged under 5 years. Int. J. Epidemiol. 38, 413–418 (2009). 18988650 10.1093/ije/dyn228

[32] Petousis-Harris H., Paynter J., Morgan J., Saxton P., McArdle B., Goodyear-Smith F., Black S., Effectiveness of a group B outer membrane vesicle meningococcal vaccine against gonorrhoea in New Zealand: A retrospective case-control study. Lancet 390, 1603–1610 (2017). 28705462 10.1016/S0140-6736(17)31449-6

[33] Alm R. A., Guerry P., Trust T. J., Distribution and polymorphism of the flagellin genes from isolates of *Campylobacter coli* and *Campylobacter jejuni*. J. Bacteriol. 175, 3051–3057 (1993). 8098328 10.1128/jb.175.10.3051-3057.1993 PMC204625

[34] Prokhorova T. A., Nielsen P. N., Petersen J., Kofoed T., Crawford J. S., Morsczeck C., Boysen A., Schrotz-King P., Novel surface polypeptides of *Campylobacter jejuni* as traveller’s diarrhoea vaccine candidates discovered by proteomics. Vaccine 24, 6446–6455 (2006). 16824653 10.1016/j.vaccine.2006.05.085

[35] Monteiro M. A., Baqar S., Hall E. R., Chen Y. H., Porter C. K., Bentzel D. E., Applebee L., Guerry P., Capsule polysaccharide conjugate vaccine against diarrheal disease caused by *Campylobacter jejuni*. Infect. Immun. 77, 1128–1136 (2009). 19114545 10.1128/IAI.01056-08 PMC2643618

[36] Burr D. H., Rollins D., Lee L. H., Pattarini D. L., Walz S. S., Tian J. H., Pace J. L., Bourgeois A. L., Walker R. I., Prevention of disease in ferrets fed an inactivated whole cell *Campylobacter jejuni* vaccine. Vaccine 23, 4315–4321 (2005). 16005742 10.1016/j.vaccine.2005.03.038

[37] Abimiku A. G., Dolby J. M., Cross-protection of infant mice against intestinal colonisation by *Campylobacter jejuni*: Importance of heat-labile serotyping (Lior) antigens. J. Med. Microbiol. 26, 265–268 (1988). 3398033 10.1099/00222615-26-4-265

[38] R. Berzofsky, in *Filtration and purification in the biopharmaceutical industry*, T. H. Meltzer, M. W. Jornitz, Eds. (Informa Healthcare, 2008), chap. 15, pp. 413–424.

[39] Hosking J., Rasanathan K., Mow F. C., Jackson C., Martin D., O’Hallahan J., Oster P., Ypma E., Reid S., Aaberge I., Crengle S., Stewart J., Lennon D., Immunogenicity, reactogenicity, and safety of a P1.7b,4 strain-specific serogroup B meningococcal vaccine given to preteens. Clin. Vaccine Immunol. 14, 1393–1399 (2007). 17898183 10.1128/CVI.00167-07 PMC2168176

[40] Lin D. Y., Dai L., Cheng G., Sailer M. O., On confidence intervals for the hazard ratio in randomized clinical trials. Biometrics 72, 1098–1102 (2016). 27123760 10.1111/biom.12528 PMC5085885

