# Supplementary material for: Vaccine-mediated protection against Campylobacter-associated enteric disease
Source: Sci Adv. 2020 Jun 24;6(26):eaba4511. doi: 10.1126/sciadv.aba4511 (PMC7314533; doi:10.1126/sciadv.aba4511)
Supplement: aba4511_SM.pdf [file aba4511_SM.pdf]

Science Advances
AAAS

advances.sciencemag.org/cgi/content/full/6/26/eaba4511/DC1

# Supplementary Materials for

## Vaccine-mediated protection against *Campylobacter*-associated enteric disease

Benjamin K. Quintel, Kamm Prongay, Anne D. Lewis, Hans-Peter Raué, Sara Hendrickson, Nicholas S. Rhoades, Ilhem Messaoudi, Lina Gao, Mark K. Slifka*, Ian J. Amanna*

*Corresponding author. Email: iamanna@najittech.com (I.J.A.); slifkam@ohsu.edu (M.K.S.)

Published 24 June 2020, *Sci. Adv.* **6**, eaba4511 (2020)
DOI: 10.1126/sciadv.aba4511

**This PDF file includes:**

Figs. S1 and S2
Tables S1 and S2

## Supplementary Materials

**Overview:**

In these studies, NHP were vaccinated against *Campylobacter* and monitored for *C. coli*- and *C. jejuni*-associated diarrhea. Diarrheal disease among rhesus macaque breeding colonies is well-documented and *Campylobacter*-associated diarrheal disease is associated with moderate to severe colitis similar to human disease (**Fig. S1**). Phylogenetic analysis, based on whole genome sequencing (WGS), was performed using a sampling of banked *Campylobacter* isolates that had been collected over a four-year period encompassing the vaccination study. Samples were tested from both asymptomatic carriers, as well as RM experiencing *C. coli*-associated diarrhea, to assess the diversity of strains found within the study population. Results included 15 *C. coli* isolates as well as the original *C. coli* vaccine strain, NTICC13 (**Fig. S2**). While serotype information cannot be directly inferred, the phylogenetic tree suggests multiple distinct strains of *Campylobacter* circulating among both asymptomatic and hospitalized animals. In spite of this degree of *Campylobacter* diversity, a single strain of the $H_2O_2$-Campy$_C$ prototype *C. coli* vaccine was able to achieve significant protective efficacy. For both vaccine cohorts, demographic comparisons were performed between vaccinated animals and contemporary, shelter-housed control animals, with no significant differences observed (**Table S1**). Comparative studies with full-length flagellin genes demonstrated high protein sequence identity among primary isolates, as well as a *C. coli* reference sequence (**Table S2**). This suggests that in contrast to the variable LOS or CPS structures found among circulating *Campylobacter* strains, it is plausible that vaccine-mediated immune responses to flagellin and/or other highly conserved bacterial surface proteins may contribute to the vaccine efficacy observed in this model.

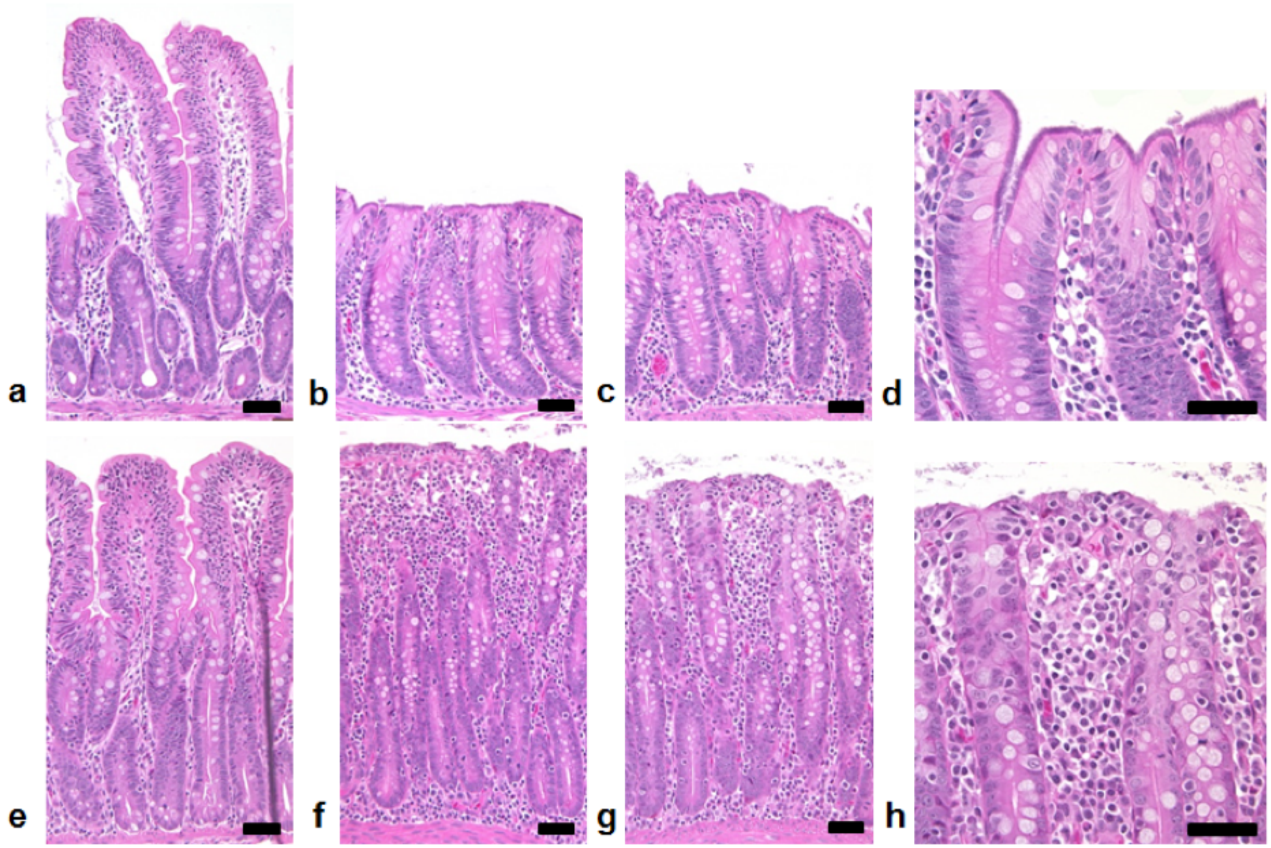


**Fig. S1. Microscopic appearance of the intestinal tract of 9-month old infant rhesus macaques with and without chronic diarrheal disease.** Top images are ileum **(a)**, cecum **(b)** and ascending colon **(c, d)** from an animal with no history of diarrhea. Bottom images are ileum **(e)**, cecum **(f)** and ascending colon **(g, h)** from an animal with multiple episodes of diarrhea, multiple fecal cultures positive for *Campylobacter coli* and linear growth stunting. The ileum is essentially the same between the normal **(a)** and diarrheic **(e)** monkeys. In contrast, the large intestine **(f, g)** of the diarrheic monkey exhibits marked mucosal hyperplasia, decreased numbers of goblet cells, and diffuse infiltration of inflammatory cells (neutrophils, lymphocytes, and plasma cells) in the lamina propria compared to the non-diarrheic monkey **(b, c)**. At higher magnification, the superficial colonic mucosa of the normal monkey **(d)** is lined by uniform tall columnar enterocytes colonized by a single population of commensal bacteria. The superficial colonic mucosa of the diarrheic monkey **(h)** is lined by short, irregular enterocytes frequently disrupted by inflammatory cells (lymphocytes and neutrophils). Hematoxylin and eosin stain, scale bar 50 microns.

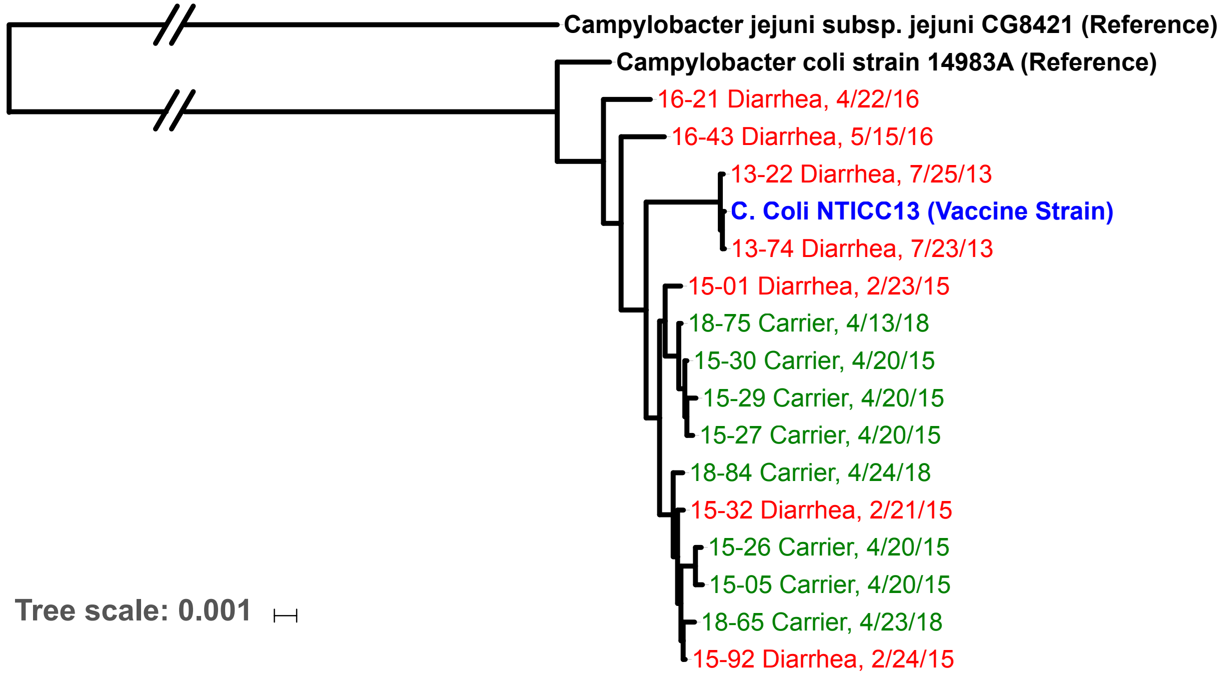


**Fig. S2. Phylogenetic analysis of *C. coli* isolates demonstrates sequence diversity within the vaccine target population.** Whole genome sequencing was performed with isolated *C. coli* cultures taken from asymptomatic carriers, as well as RM experiencing diarrheal disease. A phylogenetic tree was constructed based on a core genome alignment, using FastTree maximum likelihood via PATRICs' Phylogenetic Tree Building service (www.patricbrc.org). The tree scale is based on likelihood distance. For each isolate, the animal identification number, health status and date of isolate collection are shown. For comparison, published *C. jejuni* (GenBank: CP005388.1) and *C. coli* (GenBank: CP017025.1) reference sequences were included in the analysis. The original isolate for the *C. coli* vaccine strain (animal ID 13-22) and the final vaccine stock were also tested, demonstrating high sequence identity despite multiple rounds of sub-passage between the samples. Genome sequence accession numbers are provided in the Data and Materials Availability section.

**Table S1. Demographics of study animals**

| | 2015 Cohort | | | 2016 Cohort | | |
|---|---|---|---|---|---|---|
| | $H_2O_2$-Campy$_C$ Vaccinated[1] | $H_2O_2$-Campy$_C$ Controls[2] | *P* value[3] | $H_2O_2$-Campy$_J$ Vaccinated[1] | $H_2O_2$-Campy$_J$ Controls[2] | *P* Value[3] |
| No. Animals Monitored per Group[3] | 60 | 1645 | ----- | 67 | 1538 | ----- |
| Average Population Size per Housing Unit (range)[4] | 30 (27-33) | 36 (11-57) | 0.50 | 34 (26-41) | 43 (23-72) | 0.74 |
| Female % (no.) | 73% (44) | 61% (645) | 0.06 | 58% (39) | 62% (946) | 0.61 |
| Infant & Juvenile, <4 yr % (no.) | 70% (42) | 72% (1178) | 0.77 | 61% (41) | 71% (1090) | 0.10 |
| Adult, ≥4 yr % (no.) | 30% (18) | 28% (467) | 0.77 | 39% (26) | 29% (448) | 0.10 |

[1]Although two shelters were processed for vaccination in each vaccine cohort, these shelters included a number of non-vaccinated animals that were either temporarily unavailable at the time of vaccination or infants born after the vaccination period. These animals were included as part of the unvaccinated control animals for each cohort.

[2]For both the $H_2O_2$-Campy$_C$ and $H_2O_2$-Campy$_J$ vaccine cohorts, the remaining unvaccinated shelter-housed animals were surveyed for demographic characteristics on the first day of sheltered housing risk. Unvaccinated controls for the $H_2O_2$-Campy$_J$ vaccine study excluded $H_2O_2$-Campy$_C$ vaccinated animals from the prior year.

[3]*P* values for average size per housing unit were calculated using an unpaired, two-tailed Student's *t*-test. All other *P*-values were calculated using Fisher's exact test.

[4]To assess housing size, all shelters were surveyed at the study start date.

**Table S2. Flagellin sequence identity comparisons[1]**

**Flagellin A**

| Date of Collection | Health Status | Sample ID | NTICC13 Vaccine Strain | *C. coli* 13-74 | *C. coli* 15-29 | *C. coli* 15-30 | *C. coli* 16-21 | *C. coli* 16-43 | *C. coli* 18-75 | *C. coli* 18-84 | *C. coli* Reference[2] | *C. jejuni* CG8421[3] |
|---|---|---|---|---|---|---|---|---|---|---|---|---|
| 07.25.13 | Diarrhea | NTICC13 Vaccine Strain | 100% | 100% | 90.3% | 90.3% | 92.5% | 90.1% | 89.8% | 92.6% | 90.5% | 96.0% |
| 07.23.13 | Diarrhea | *C. coli* 13-74 | | 100% | 90.3% | 90.3% | 92.5% | 90.1% | 89.8% | 92.6% | 90.5% | 96.0% |
| 04.20.15 | Carrier | *C. coli* 15-29 | | | 100% | 100% | 89.4% | 99.0% | 98.8% | 89.6% | 99.8% | 89.4% |
| 04.20.15 | Carrier | *C. coli* 15-30 | | | | 100% | 89.4% | 99.0% | 98.8% | 89.6% | 99.8% | 89.4% |
| 04.22.16 | Diarrhea | *C. coli* 16-21 | | | | | 100% | 89.6% | 89.1% | 91.0% | 89.4% | 93.7% |
| 05.15.16 | Diarrhea | *C. coli* 16-43 | | | | | | 100% | 98.8% | 89.6% | 98.8% | 89.4% |
| 04.13.18 | Carrier | *C. coli* 18-75 | | | | | | | 100% | 88.9% | 98.6% | 88.9% |
| 04.24.18 | Carrier | *C. coli* 18-84 | | | | | | | | 100% | 89.6% | 91.3% |
| -------- | -------- | *C. coli* Reference | | | | | | | | | 100% | 89.6% |
| -------- | -------- | *C. jejuni* CG8421 | | | | | | | | | | 100% |

**Flagellin B**

| Date of Collection | Health Status | Sample ID | NTICC13 Vaccine Strain | *C. coli* 13-74 | *C. coli* 15-29 | *C. coli* 15-30 | *C. coli* 16-21 | *C. coli* 16-43 | *C. coli* 18-75 | *C. coli* 18-84 | *C. coli* Reference[2] | *C. jejuni* CG8421[3] |
|---|---|---|---|---|---|---|---|---|---|---|---|---|
| 07.25.13 | Diarrhea | NTICC13 Vaccine Strain | 100% | 100% | 89.4% | 89.4% | 92.7% | 89.4% | 89.1% | 92.9% | 89.3% | 97.0% |
| 07.23.13 | Diarrhea | *C. coli* 13-74 | | 100% | 89.4% | 89.4% | 92.7% | 89.4% | 89.1% | 92.9% | 89.3% | 97.0% |
| 04.20.15 | Carrier | *C. coli* 15-29 | | | 100% | 100% | 88.9% | 97.6% | 98.1% | 89.8% | 98.6% | 88.9% |
| 04.20.15 | Carrier | *C. coli* 15-30 | | | | 100% | 88.9% | 97.6% | 98.1% | 89.8% | 98.6% | 88.9% |
| 04.22.16 | Diarrhea | *C. coli* 16-21 | | | | | 100% | 89.8% | 89.2% | 92.2% | 89.1% | 94.8% |
| 05.15.16 | Diarrhea | *C. coli* 16-43 | | | | | | 100% | 99.5% | 89.9% | 99.0% | 89.3% |
| 04.13.18 | Carrier | *C. coli* 18-75 | | | | | | | 100% | 90.1% | 99.5% | 88.9% |
| 04.24.18 | Carrier | *C. coli* 18-84 | | | | | | | | 100% | 90.1% | 91.3% |
| -------- | -------- | *C. coli* Reference | | | | | | | | | 100% | 88.9% |
| -------- | -------- | *C. jejuni* CG8421 | | | | | | | | | | 100% |

[1]Amino acid percent identity comparisons were performed for isolates with complete *flaA* or *flaB* gene sequences. Genome sequence accession numbers are provided in the Data and Materials Availability section.

[2]GenBank: CP017025.1, locus tags CC14983A_0418 (*flaA*) and CC14983A_0419 (*flaB*)

[3]GenBank: CP005388.1, locus tags CJ8421_06685 (*flaA*) and CJ8421_06680 (*flaB*)
